# Application of chimeric flaps for complex orbitomaxillary reconstruction in sinonasal malignancy

**DOI:** 10.3389/fonc.2026.1963043

**Published:** 2026-10-23

**Authors:** Karnav Panchal, Kapila Manikantan, Saugata Samadder, P. Arun, Vikram D. Kekatpure, Prateek V. Jain, Gurucharan Sahu

**Affiliations:** 1 Department of Plastic, Reconstructive Surgery, Tata Medical Center Kolkata, Kolkata, India; 2 Department of Head and Neck Surgery, Tata Medical Center Kolkata, Kolkata, India

**Keywords:** ALT flap, chimeric flaps, fibula flap, scapula flap, sinonasal malignances

## Abstract

**Background:**

Sinonasal malignancies are rare tumors, and a subset of these patients presents with involvement of the orbitomaxillary area, resulting in a complex maxillectomy defect, which warrants primary reconstruction. Complexity increases with globe preservation, which mandates structural support to the globe, ideally reconstructed using a vascularized osseous flap. Such complex defect reconstruction requires multiple components, which are titrated according to the complex three-dimensional defects.

**Objectives:**

This study aims to evaluate the outcomes of primary orbitomaxillary reconstruction in sinonasal malignancy using multicomponent chimeric flaps, which included osseous chimeric flaps for globe-preserving total maxillectomy and soft tissue chimeric flaps for the remaining orbitomaxillary defects.

**Materials and methods:**

A retrospective observational study was conducted between June 2024 and April 2026 at a tertiary referral center. The cohort included consecutive patients with modified Brown type 3 to 6 maxillectomy defects having sinonasal malignancy, which were primarily reconstructed with chimeric flaps. Data on the pathology, type of orbito maxillectomy defects, and types and components of chimeric flaps were collected. Outcomes were measured by flap survival, re-explorations, wound healing compromise, and delays in the start of adjuvant therapy. Specifically for orbital preservation patients, further assessment was made on the presence of diplopia or enophthalmos.

**Results:**

All 11 patients included in the study underwent a chimeric flap for orbitomaxillary defects. The most common pathology was squamous cell carcinoma. Seven (64%) were soft tissue chimeric flaps, and four (36%) were osseous chimeric flaps. All flaps survived completely. Satisfactory wound healing and contour restoration were achieved in all patients.

**Conclusion:**

Chimeric flaps with their multiple components, which can be contoured according to the complex three-dimensional orbitomaxillary defects, achieves primary healing. For total maxillectomy with globe preservation, bone reconstruction is warranted. Orbital bone reconstruction use of 3D model for contouring the bone strut and the orbital floor mesh can achieve accurate reconstruction for such complex defects.

## Introduction

Sinonasal malignancies are heterogeneous but rare subsets of cancer accounting for 3%–5% of all head and neck cancers (1). Sinonasal tumors are mostly either epithelial (carcinoma) or mesothelial (sarcoma) in origin. Surgery is the mainstay of treatment for advanced sinonasal tumors, with radiotherapy and chemotherapy used as adjuvant treatment modalities (2).

Early attempts at maxillary reconstruction date back to the first description of maxillectomy. In the sixteenth century, French surgeon Ambroise Pare designed an “obturateur” to address acquired palatal defects secondary to battlefield injuries in the military (3). In the early eighteenth century, French dentist Pierre Fauchard published a review of obturator devices for maxillectomy defects (3). In the late eighteenth century, the first tooth-supported obturator was proposed by dental surgeon Bernard Bourdet. Maxillary reconstruction was born in military medicine and evolved from prosthetics to complex flaps and incorporation of computer-aided 3D planning that we use today (4).

After surgical resection, appropriate primary reconstruction should be planned with the intent to close oronasal communication, seal dural defects, restore the contour of the maxillofacial skeleton, and most importantly support the globe to avoid late complications like enophthalmos, providing dental rehabilitation and minimizing the complications of post-operative RT. The concept of chimeric flap was introduced by Hallock et al. as a single flap consisting of multiple components having different tissue compositions but a single vascular pedicle (5). Chimeric flaps aid in component-based reconstruction for complex defects.

## Materials and methods

This is a retrospective hospital-based study from June 2024 to April 2026. The inclusion criteria were all patients with modified Brown type 3 to 6 maxillectomy defect which were reconstructed with chimeric flaps primarily. The exclusion criteria were primary type 1 and 2 maxillectomy defects and secondary defects planned for reconstruction. Isolated orbital exenteration defects without a maxillectomy were also excluded from the study.

Preoperatively, all patients underwent radiological evaluations by MRI and CT scan. The reconstruction procedure was performed at the same time as the tumor removal. Data on the type of maxillectomy, pathology, and stage of malignancy were collected. Data on the type and components of chimeric flap utilized and the recipient vessels were retrieved.

For patients with orbital exenteration, a chimeric soft tissue flap was planned, and the flap of choice was anterolateral thigh flap. For patients planned for total maxillectomy with globe preservation, bone flaps were planned, and 3D skull models were fabricated from preoperative CT scans. These models served as templates to contour the structural bony struts and the titanium meshes for orbital floor reconstruction. The flap of choice was a fibula osteocutaneous chimeric flap. However, one patient not willing for fibula flap was reconstructed with a chimeric scapula flap. A 3D model was prepared from the CT scan sections of all patients who were planned for bone reconstruction. Based on the model, the bone segment requirements were determined, and accurate contouring of the orbital floor mesh was performed intraoperatively.

Immediate outcomes were evaluated based on any vascular compromise in the flap and any flap failure. Early-healing-related complications, such as wound dehiscence, marginal flap necrosis, seroma, or fistula formation, were assessed. For patients who underwent total maxillectomy with globe preservation, outcomes were measured with the presence or absence of diplopia and enophthalmos.

## Result

A total of 11 patients were included in the study design. Eight (72%) were men, and three (28%) were women. The mean age was 54 years (28–76 years). The tumor pathology was recurrent in seven patients (64%), and only four patients (36%) presented with primary tumors. Out of the seven patients with recurrent tumors, four of them were operated on multiple times. Out of the four primary tumors, two received neoadjuvant chemotherapy for downstaging. The pathology was squamous cell carcinoma in five patients (45%) and adenoid cystic carcinoma in three patients (28%). Two patients (18%) had sarcoma and one patient (9%) had high-grade neuroblastoma (**Table 1**).

**Table 1:** Demographic data, pathology, maxillectomy defect, stage, and the excision margin status.

Sr No	Age/Sex	Pathology	Maxillectomy browns	Stage	Excision margin status
1	28/F	Primary Alveolar RMS	Type 5	T4N0	R0
2	76/M	Recurrent SCC	Type 5	T4N0	R1
3	69/F	Primary SCC	Type 5	T3N0	R0
4	63/M	Re-recurrent Adenoid Cystic Ca	Type 5	T4No	R0
5	64//M	Re-recurrent Extraskeletal chondrosarcoma	Type 5	T4N0	R0
6	59/M	Primary Adenoid Cystic Ca	Type 4	T4N0	R0
7	42/M	Primary Olfactory neuroblastoma	Type 6	T3N0	R1
8	34/M	Recurrent SCC	Type 3c	T3No	R0
9	58/M	Re-recurrent Basosqumous carcinoma	Type 3c + Cheek skin + Nose	T4No	R0
10	50/F	Recurrent Adenoid cystic Ca	Type 3c	T3NO	R0
11	58/M	Recurrent Sinonasal Carcinoma	Type 3C + posterior mandibulectomy	T2N0	R0

Based on the modified Brown classification, four patients (36%) had type 3 total maxillectomy defects with the orbital contents preserved. One patient had type 4 and type 6 defects. The most common type was type 5 defects in five patients (45%). Following tumor excision, we performed immediate primary reconstruction using a chimeric flap for all patients. For types 4, 5, and 6 defects, soft tissue reconstruction was done, which accounted for seven patients. A chimeric ALT flap was used in six patients, and one patient had a chimeric LD flap. All four type 3 defect patients had primary bone reconstruction with a chimeric osseous flap. Three of them were chimeric fibula flap and one was chimeric scapula flap. One patient required an additional radial forearm flap to reconstruct the external skin defect as well (**Table 2**).

**Table 2:** Type of flaps, chimeric flap composition, recipient vessels selected, and the adjuvant therapy data.

Sr No	Flap	Chimera components	Recipent vessels	Adjuvant therapy
1	Chimeric ALT	3 components : 3 skin	Facial Vessels	RT+ CT
2	Chimeric ALT	3 components : 1 skin , 2 muscle	Facial	RT
3	Chimeric ALT	2 components 1 skin , 1 muscle	Facial Vessels	RT
4	Chimeric LD	2 components : 1 skin , 1 muscle	RAFF vessels done earlier	RT
5	Chimeric ALT	2 components : 1 skin , 1 muscle	Facial vessels	RT
6	Chimeric ALT	3 components : 2 skin 1 muscle	Facial Vessels	RT
7	Chimeric ALT	4 components : 2 skin , 1 muscle , 1 vascularised fascia	Superficial temporal vessels	RT
8	Chimeric Scapula	3 components : 2 bone , 1 skin muscle	Facial	RT
9	Chimeric Fibula + RAFF	4 components :Fibula : 2 bone 2 skin + RAFF	Facial vessels , RAFF intrinsic fabrication	RT
10	Chimeric Fibula	4 components : 2 bone , 2 skin	Facial vessels	RT
11	Chimeric Fibula	4 components : 2bone 1 skin 1 muscle	Facial vessels	No

As per the concept of chimeric flaps, each component of the flap was based on an independent arterial input and venous drainage. Component-wise reconstruction helped in better adaptability of the flap to the three-dimensional defect created. Four patients had four components, another four patients had three components of the chimeric flap, and three patients had two components. The most common recipient vessels were the facial vessels, which were used in nine patients (81%); superficial temporal vessels were used in one patient, and in one recurrent patient the previous flap pedicle was used as the recipient vessels. There were no flap re-explorations, and all of the flaps survived. The mean duration of hospital stay was nine days (range, 7–14 days). Except for the two fibula donor sites, all other nine donor sites were closed primarily. There were no donor-site-related complications.

All of the flaps had satisfactory healing, except for one chimeric ALT flap which developed marginal skin necrosis and superficial infection that required an extra course of antibiotics and which healed with regular dressing. A patient of craniofacial resection had a CSF leak, which healed conservatively. No patient developed seroma, fistula formation, or dehiscence.

Out of the 11 patients, R0 resection was achieved in 10 patients, and one patient had R1 resection and received adjuvant radiotherapy. A total of 10 (91%) patients received adjuvant radiotherapy. All 10 patients initiated radiotherapy from 4 to 7 weeks post-surgery (mean, 5.6 weeks). There was no delay in the initiation of adjuvant therapy in any of the patients due to wound-related issues. All patients were followed up for a minimum of 3 months post-surgery. Mean follow-up was 8 months (range, 3–18 months). Out of the four patients requiring bone chimeric flaps, no patient developed enophthalmos or diplopia. Out of the patients with orbital exenteration, two (40%) patients were rehabilitated with spectacle-mounted prosthesis. Other patients did not opt for any orbital prosthesis rehabilitation.

Case 1: A 60 year-old gentleman had a recurrent extra-skeletal chondrosarcoma involving the right orbit. He was operated on twice and had a recurrence 9 years later. The surgery resulted in a type 5 maxillectomy defect. Reconstruction was done with a chimeric ALT flap. The patient received adjuvant radiotherapy. Post-operative photographs taken 12 months after the completion of treatment are shown in **Figures 1**–**4**.

**Figure 1:**
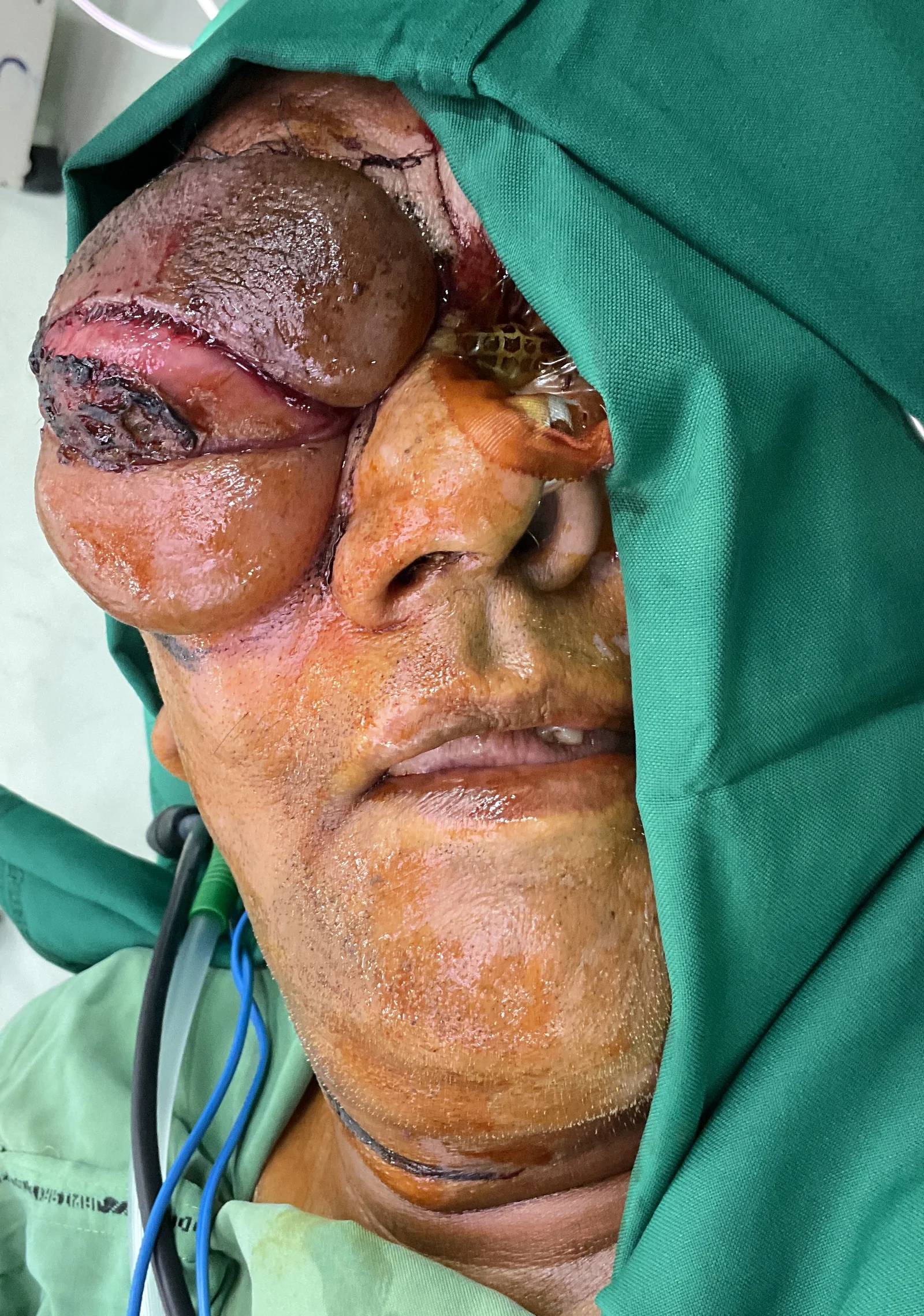
Lateral view pre-excision.

**Figure 2:**
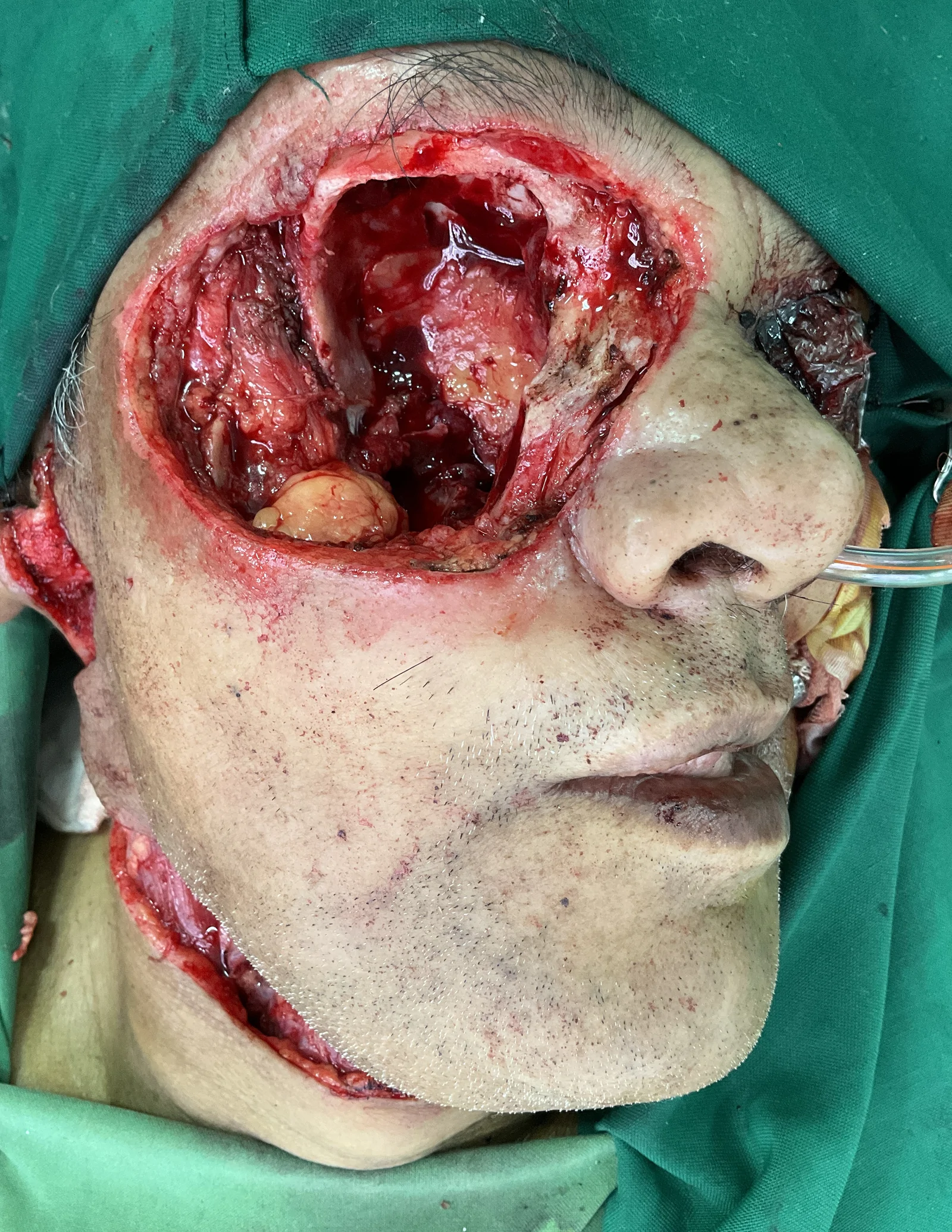
Defect post-excision anterior view.

**Figure 3:**
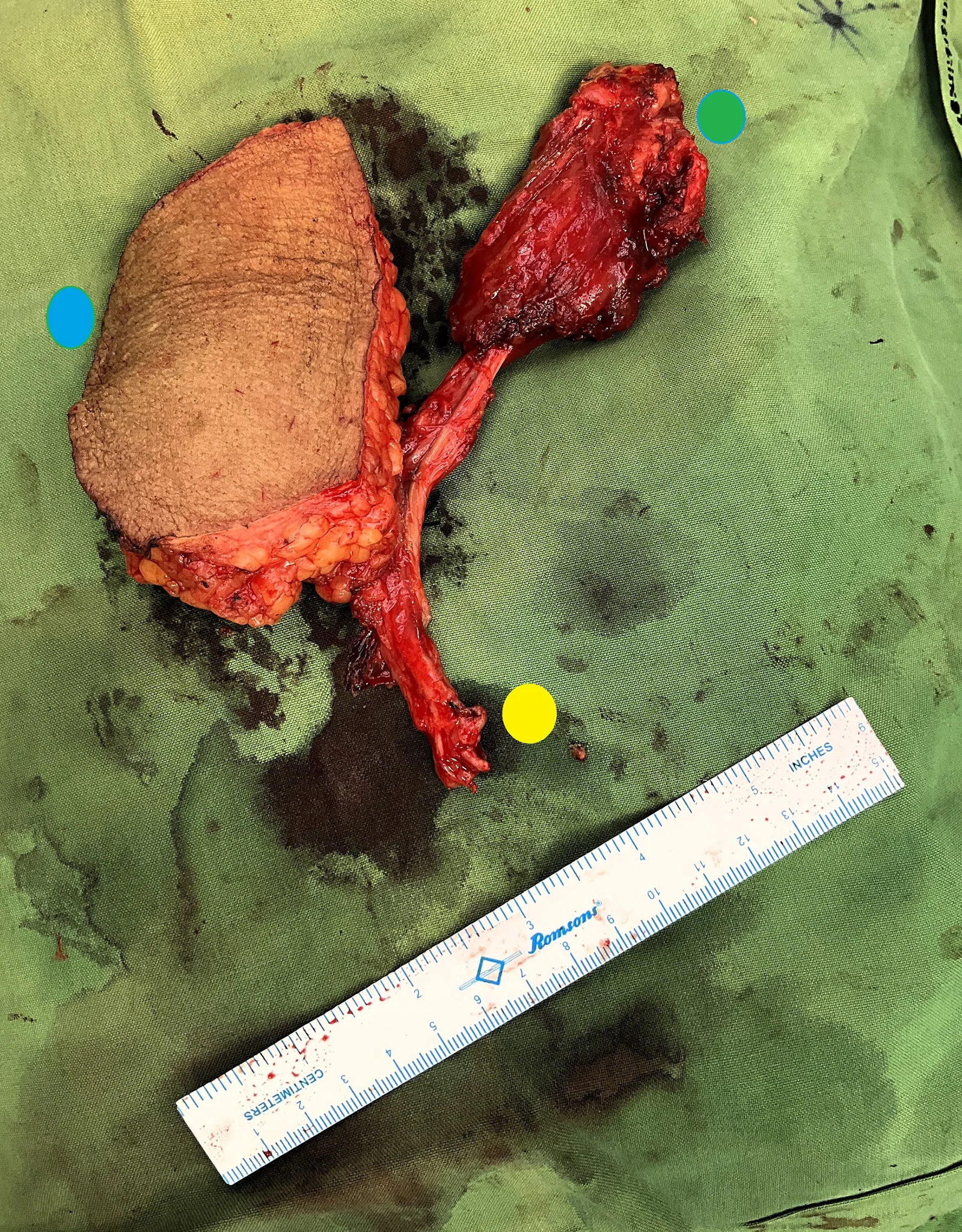
Chimeric ALT flap. Yellow dot, descending LCFA pedicle; blue dot, skin island based on a perforator; green dot, vastus lateralis muscle based on the distal runoff of vessel.

**Figure 4:**
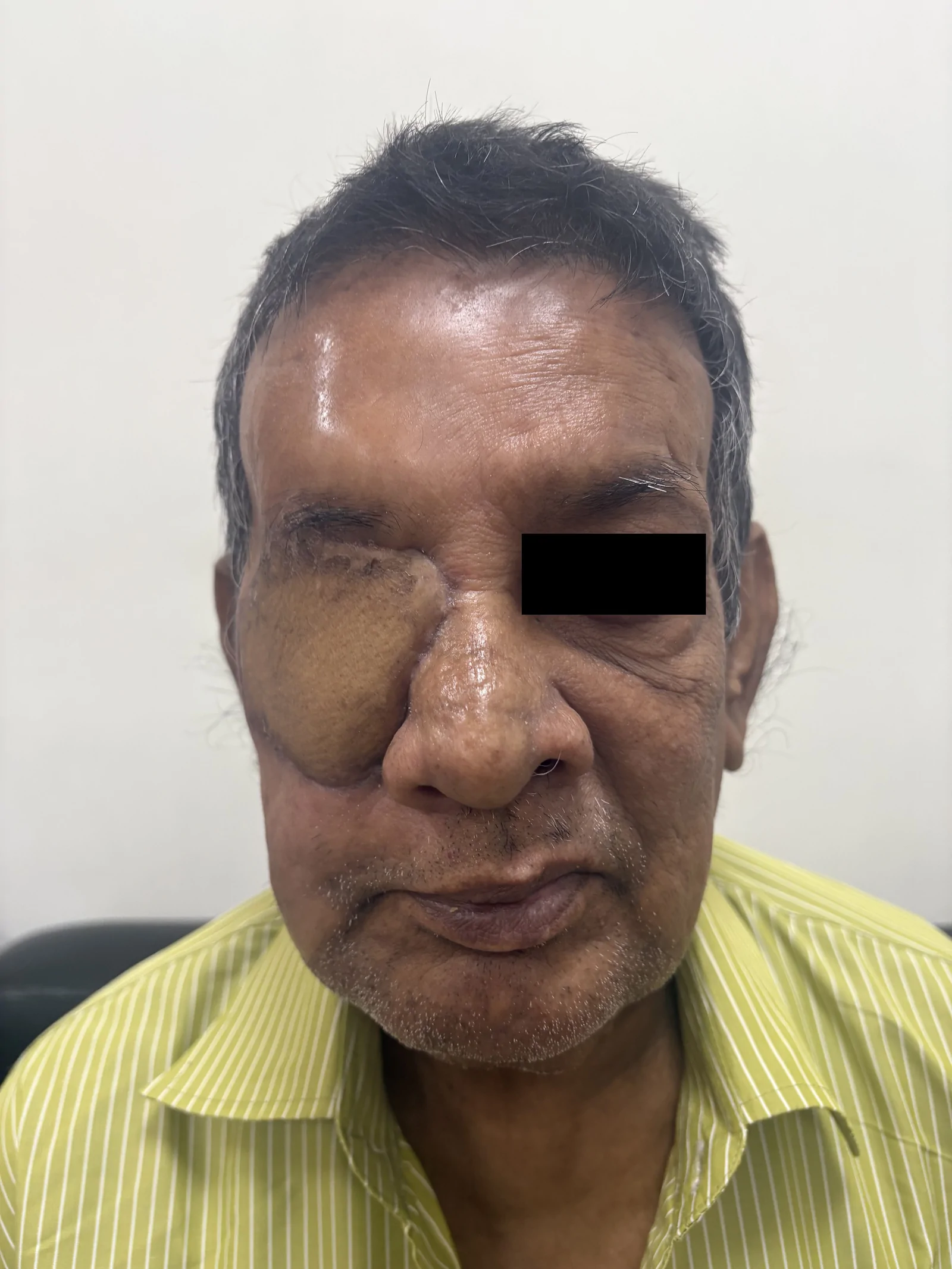
Post-operative anterior view.

Case 2: A 34-year-old gentleman with squamous cell carcinoma underwent right-sided total maxillectomy. Reconstruction was done with a fabricated chimeric scapula flap. The scapular angle and lateral border were supplied by the angular branch arising from the circumflex scapular artery. Based on the thoracodorsal pedicle, the LD muscle and skin components were harvested. The flap fabrication was done by anastomosing the artery and vein of the circumflex pedicle to the serratus branch. The main thoracodorsal pedicle was anastomosed to the facial vessels. This fabricated chimeric flap had four components: the angle of the scapula was used to reconstruct the orbital floor and infraorbital rim, the lateral border of the scapula was used to reconstruct the upper alveolus, the LD muscle was used to reconstruct the lining, and the skin was used to reconstruct the nasal lining. Postoperative photographs taken 3 months post-surgery show adequate structural support to the eye and good shoulder function (**Figures 5**–**11**).

**Figure 5:**
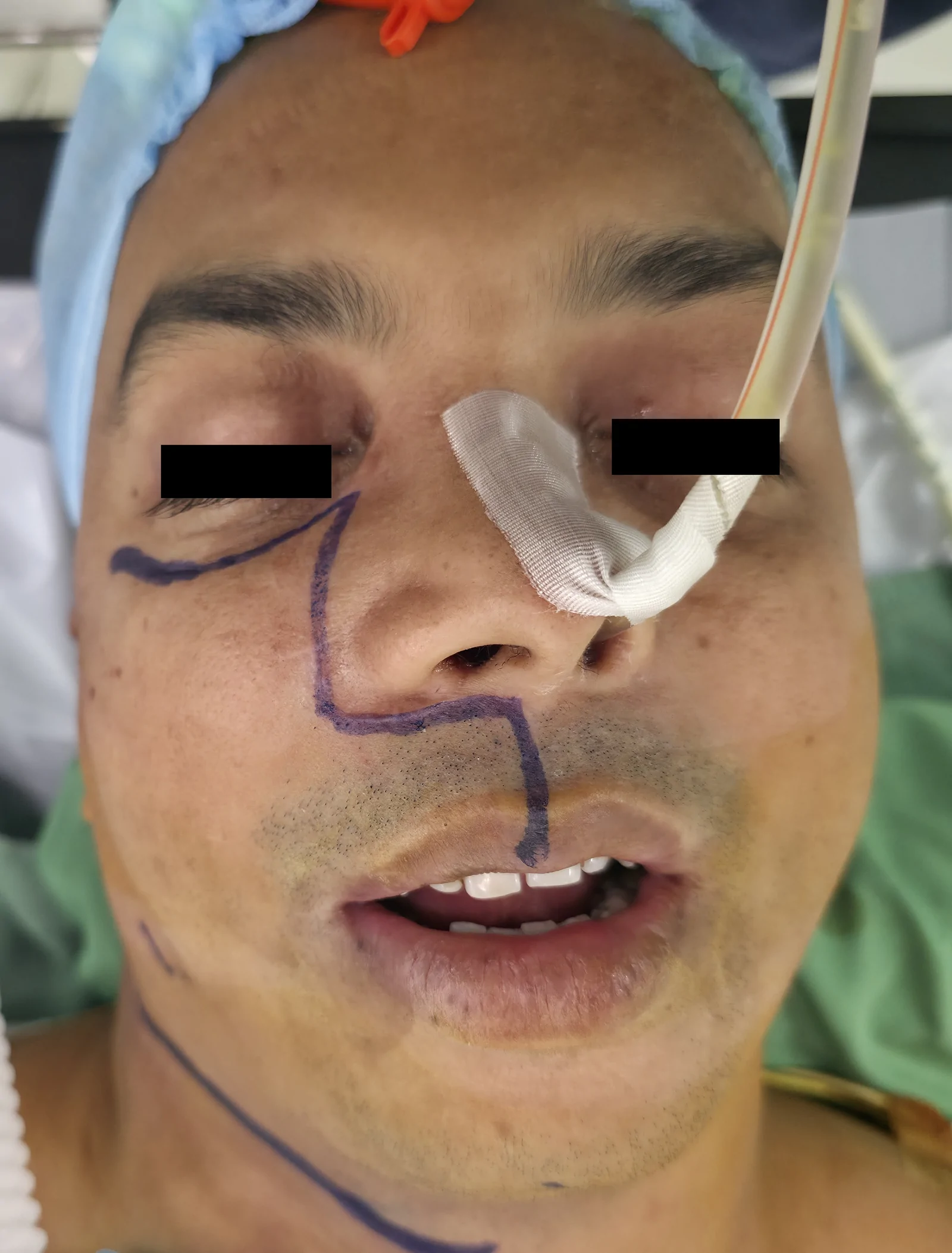
Pre-surgery view.

**Figure 6:**
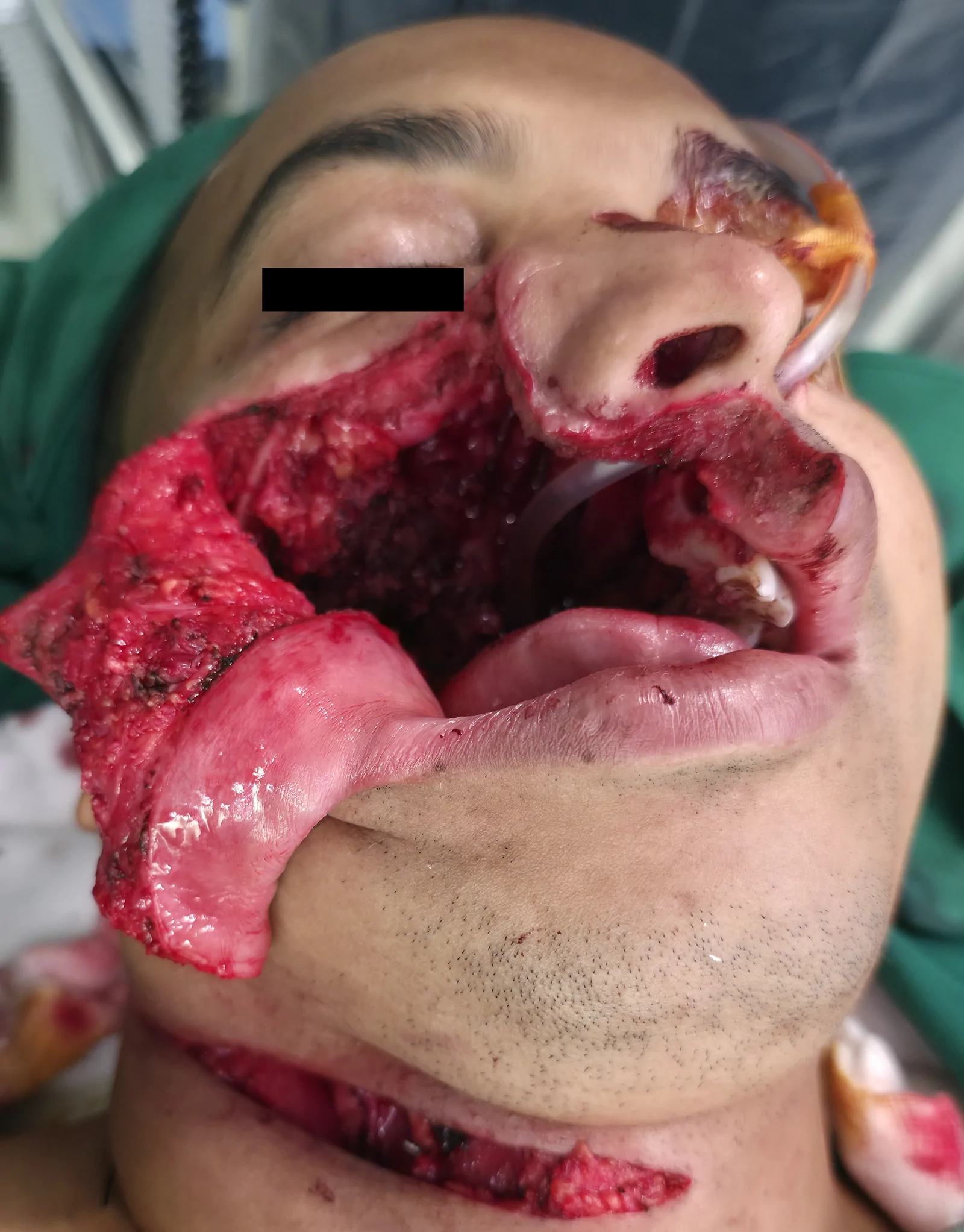
Total maxillectomy defect.

**Figure 7:**
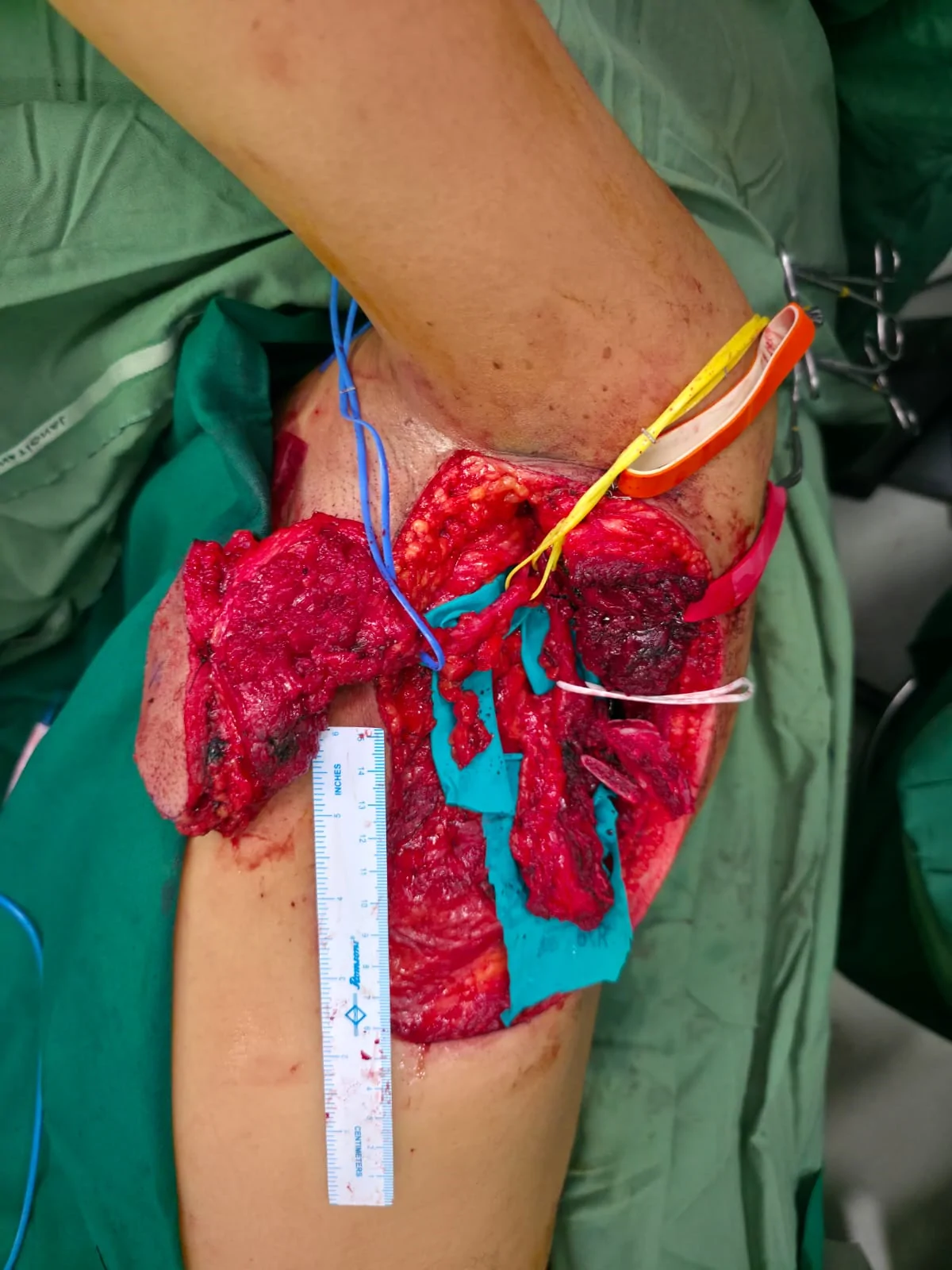
Chimeric scapula flap harvest in lateral position.

**Figure 8:**
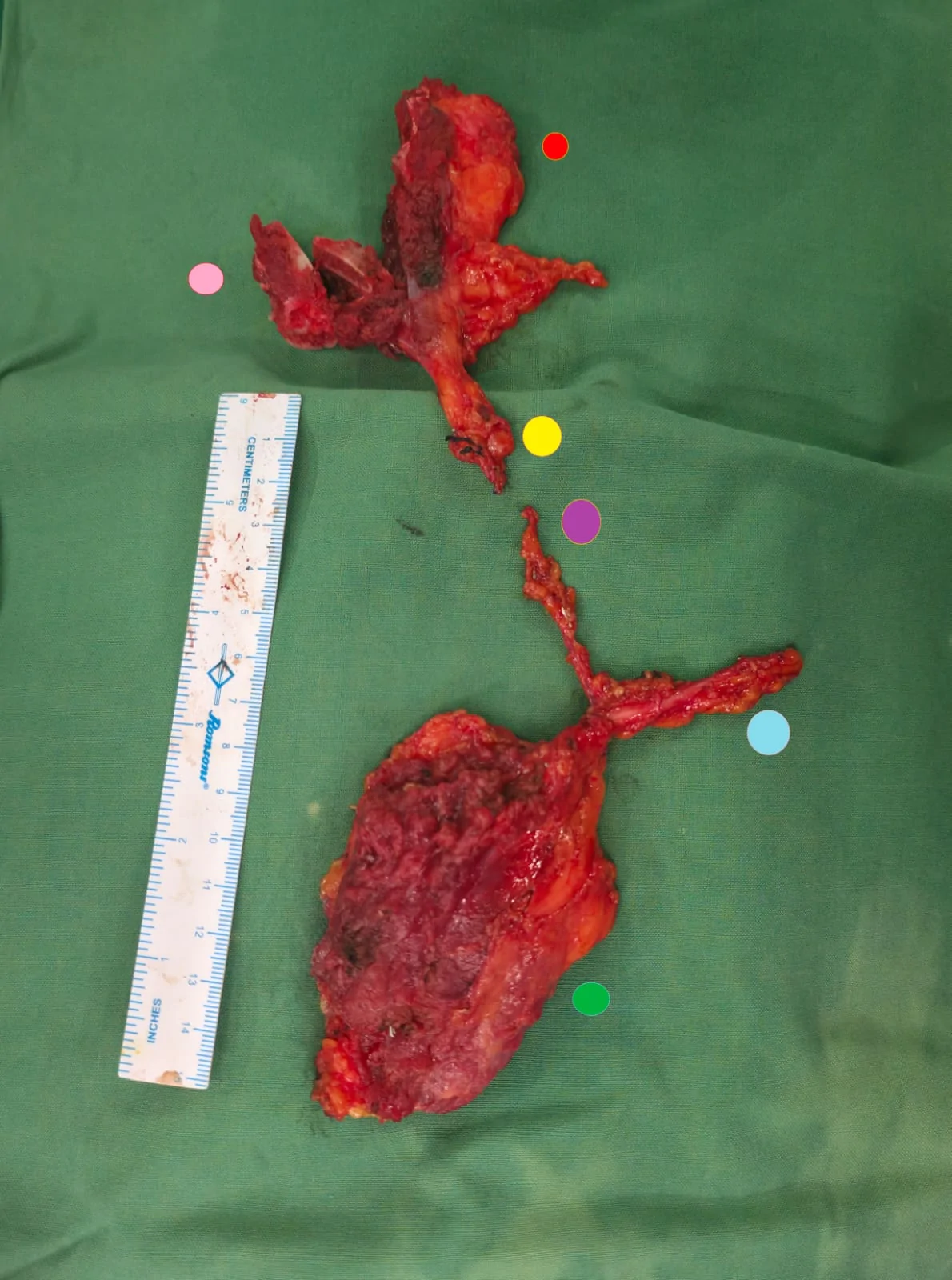
Chimeric scapula flap, would require anastomosis for fabrication. Red dot, medial border of scapula used for maxillary alveolar bone; pink dot, angular bone of scapula used for orbital floor; green dot, LD muscle for lining and filling up antrum; blue dot, thoracodorsal pedicle; yellow dot, angular branch which was joined to the branch to serratus represented by the purple dot.

**Figure 9:**
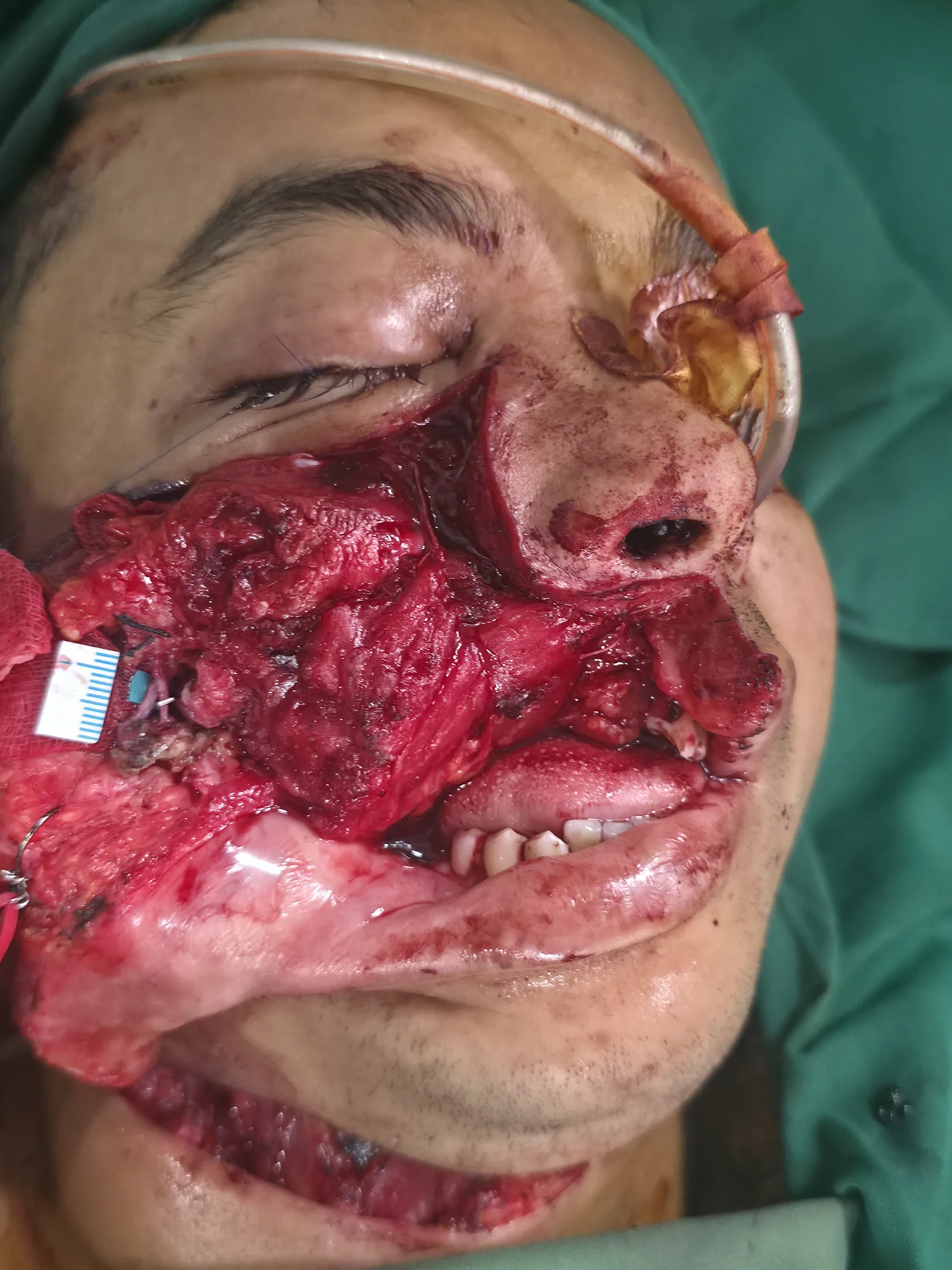
Anastomosis between circumflex scapular vessels to serratus branch vessels.

**Figure 10:**
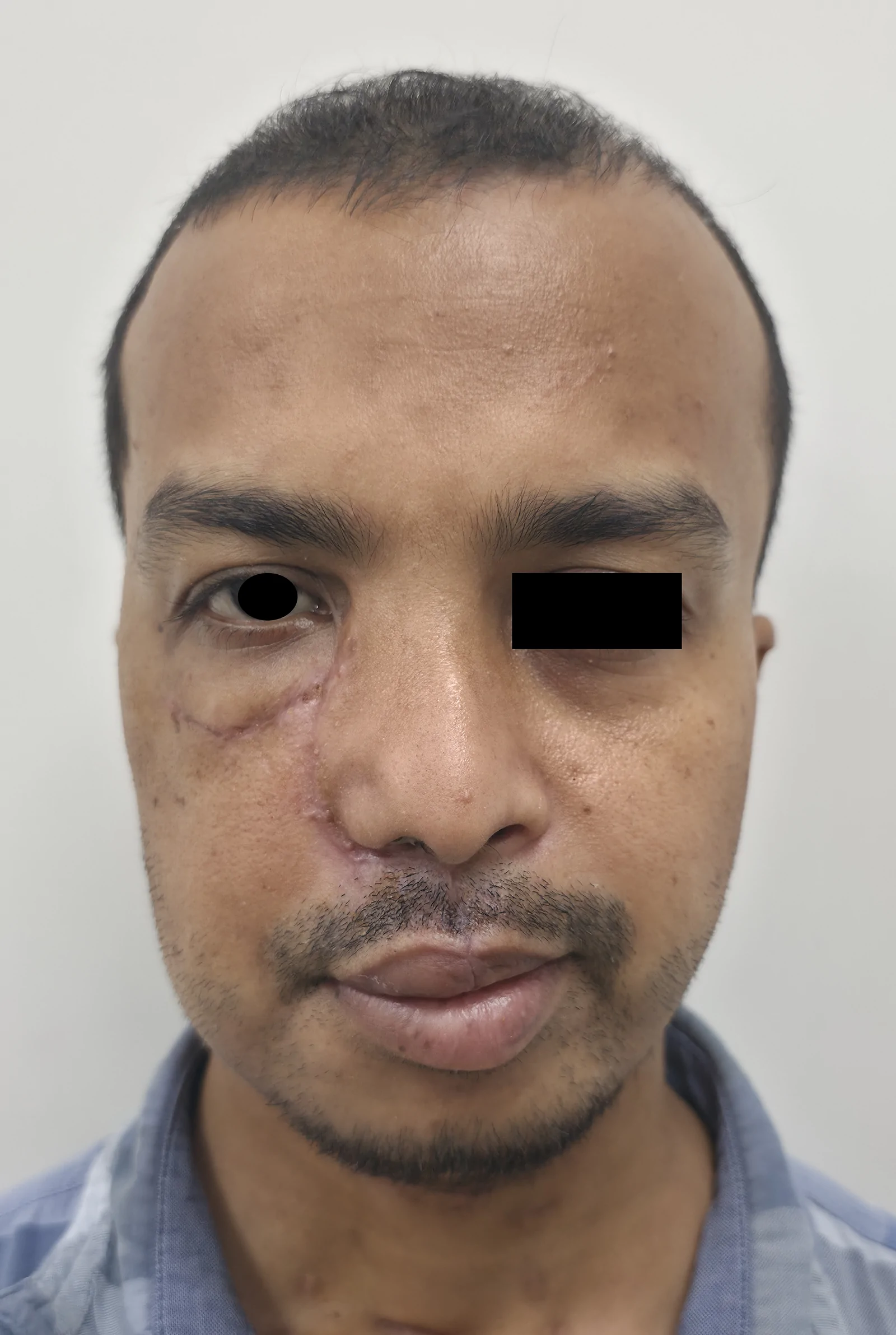
Post-operative result.

**Figure 11:**
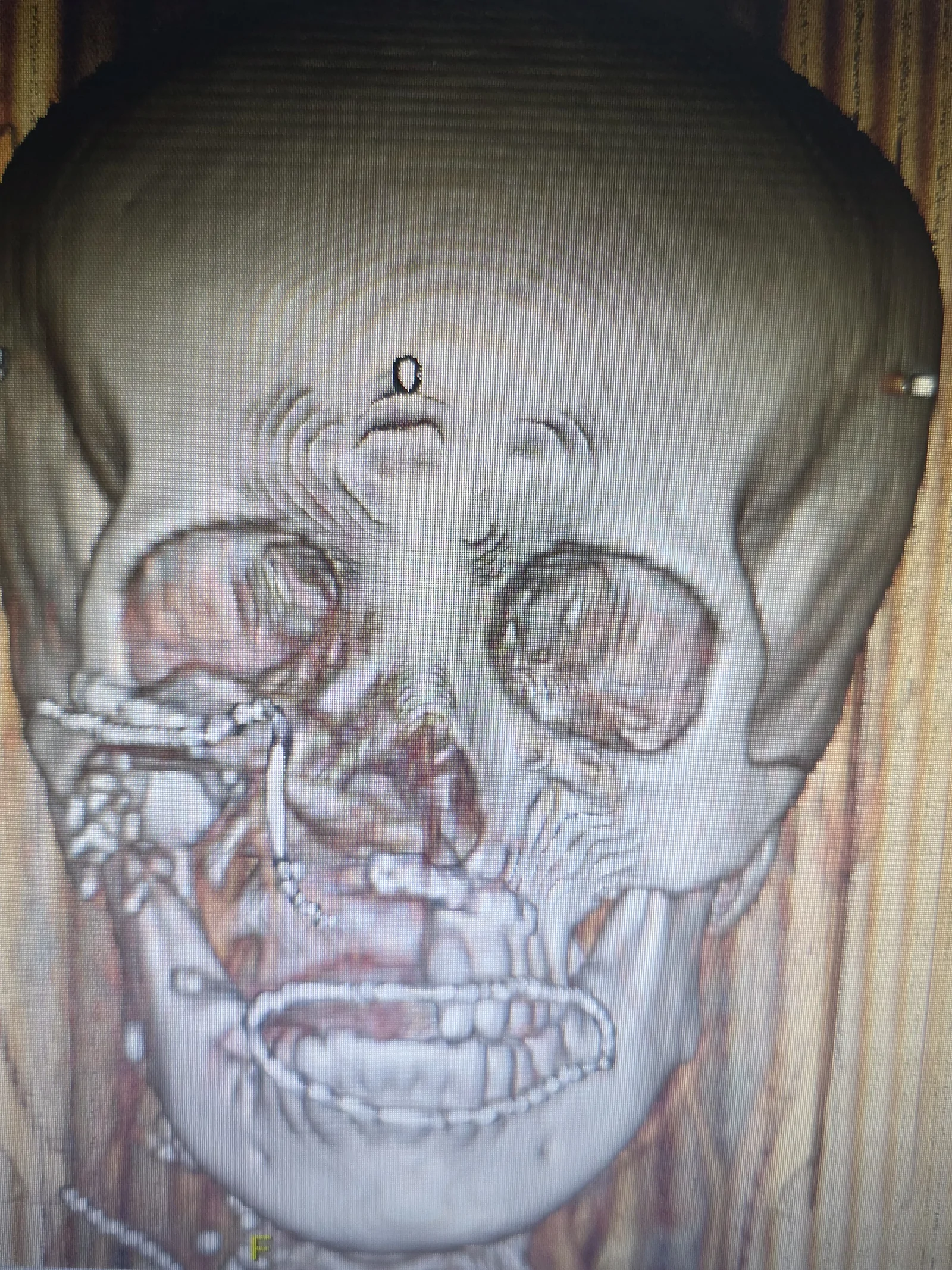
3D CT scan post-operatively.

Case 3: A 50-year-old lady had a recurrent adenoid cystic carcinoma; she underwent a right-sided total maxillectomy through the previous surgical incision. Reconstruction was done with a chimeric fibula flap. A 3D model was used to design the bone segments as well as to contour the titanium mesh for the orbital floor reconstruction. The intrinsic chimeric fibula flap had four components. Two bone struts were used, one for the infraorbital rim and one for the upper alveolus. Two skin islands were used, one for the nasal lining and one for the palatal lining. Primary skin closure of the donor site was achieved. The patient had an uneventful recovery (**Figures 12**–**15**).

**Figure 12:**
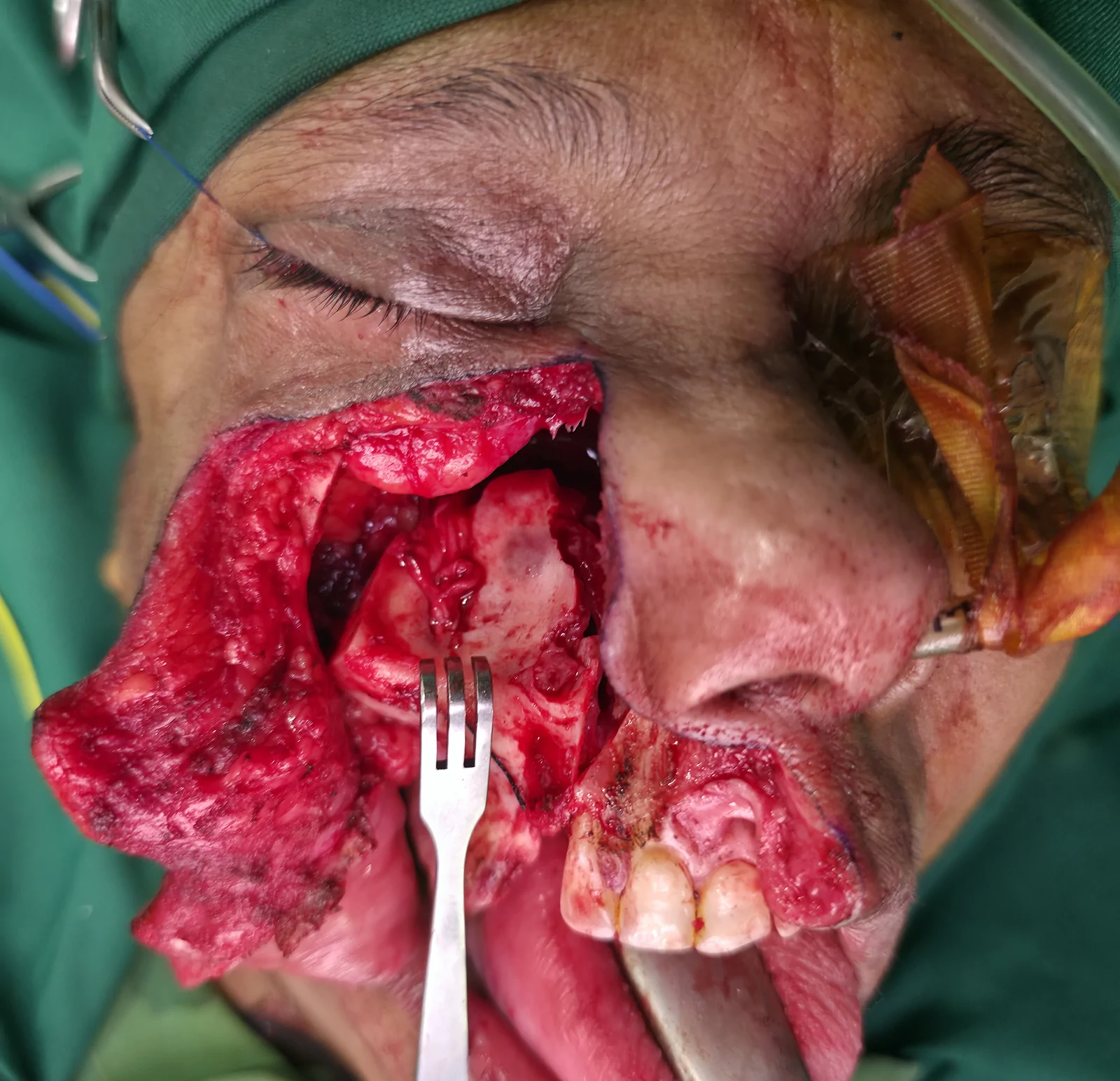
Total maxillectomy intraoperative view.

**Figure 13:**
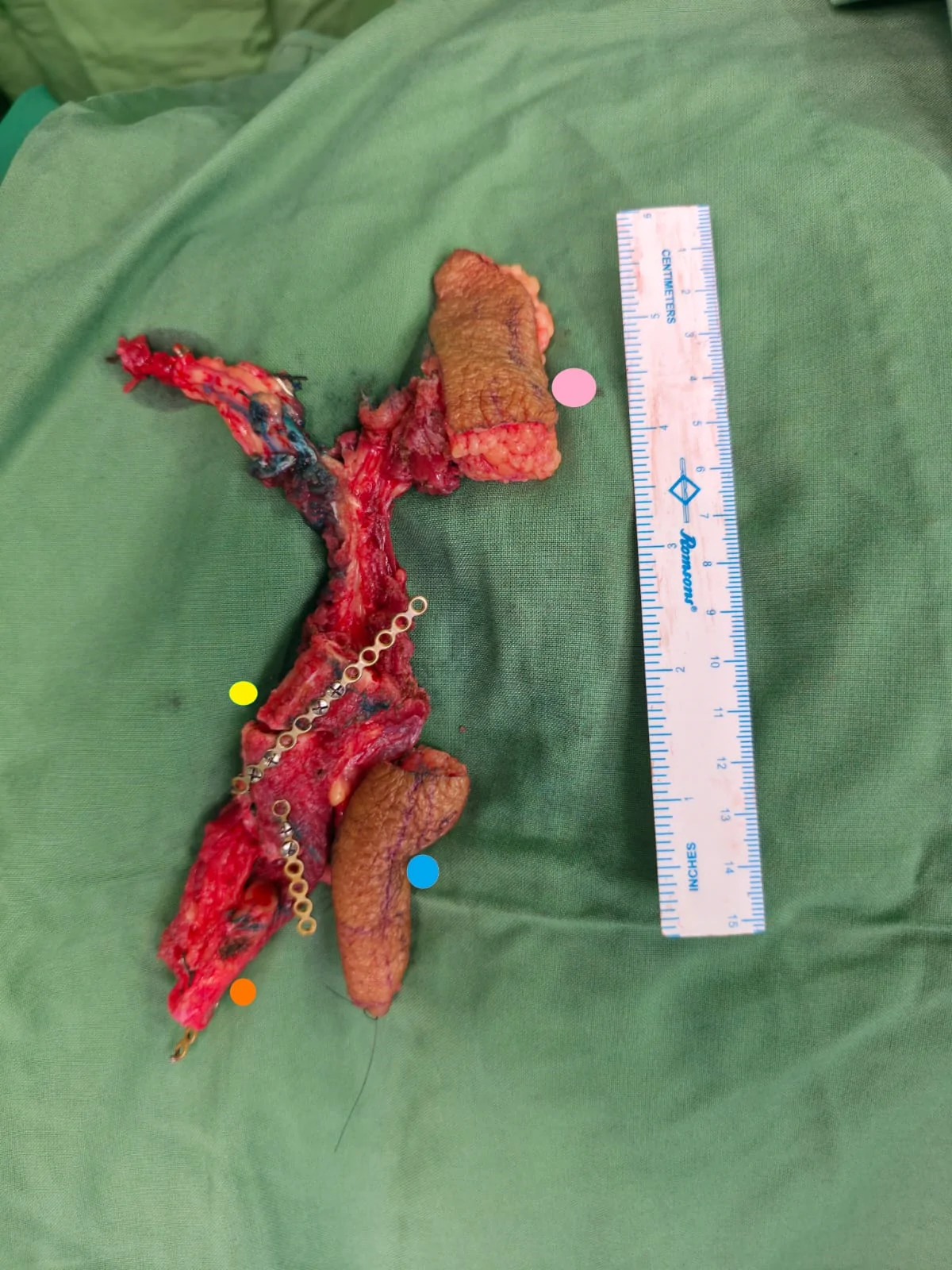
Chimeric fibula flap. Orange dot, fibula bone for infraorbital rim; yellow dot, fibula bone for maxilla alveolar bone; blue dot, skin island for nasal lining; pink dot, skin island for palatal lining.

**Figure 14:**
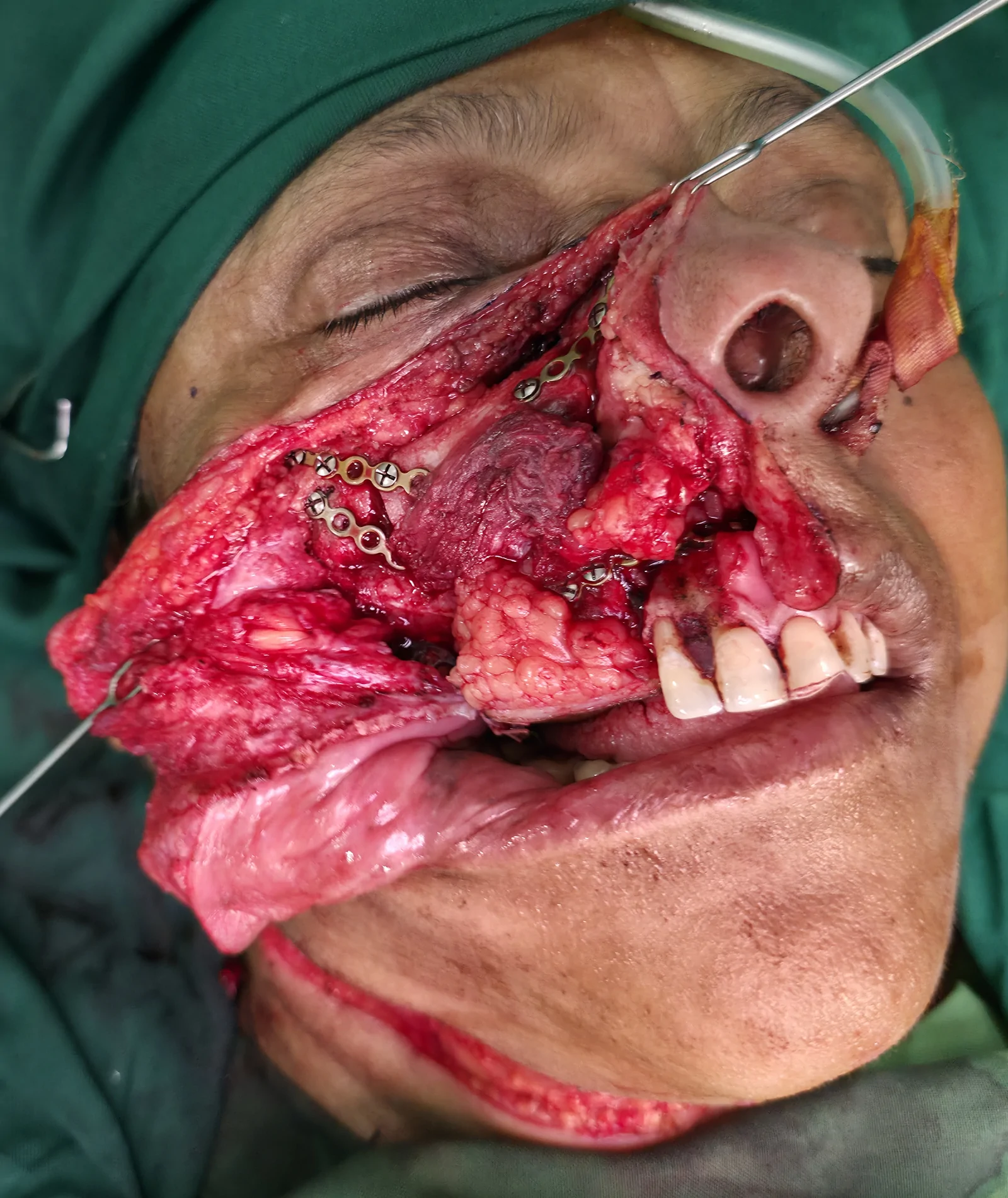
Intraoperative bone and skin fixation.

**Figure 15:**
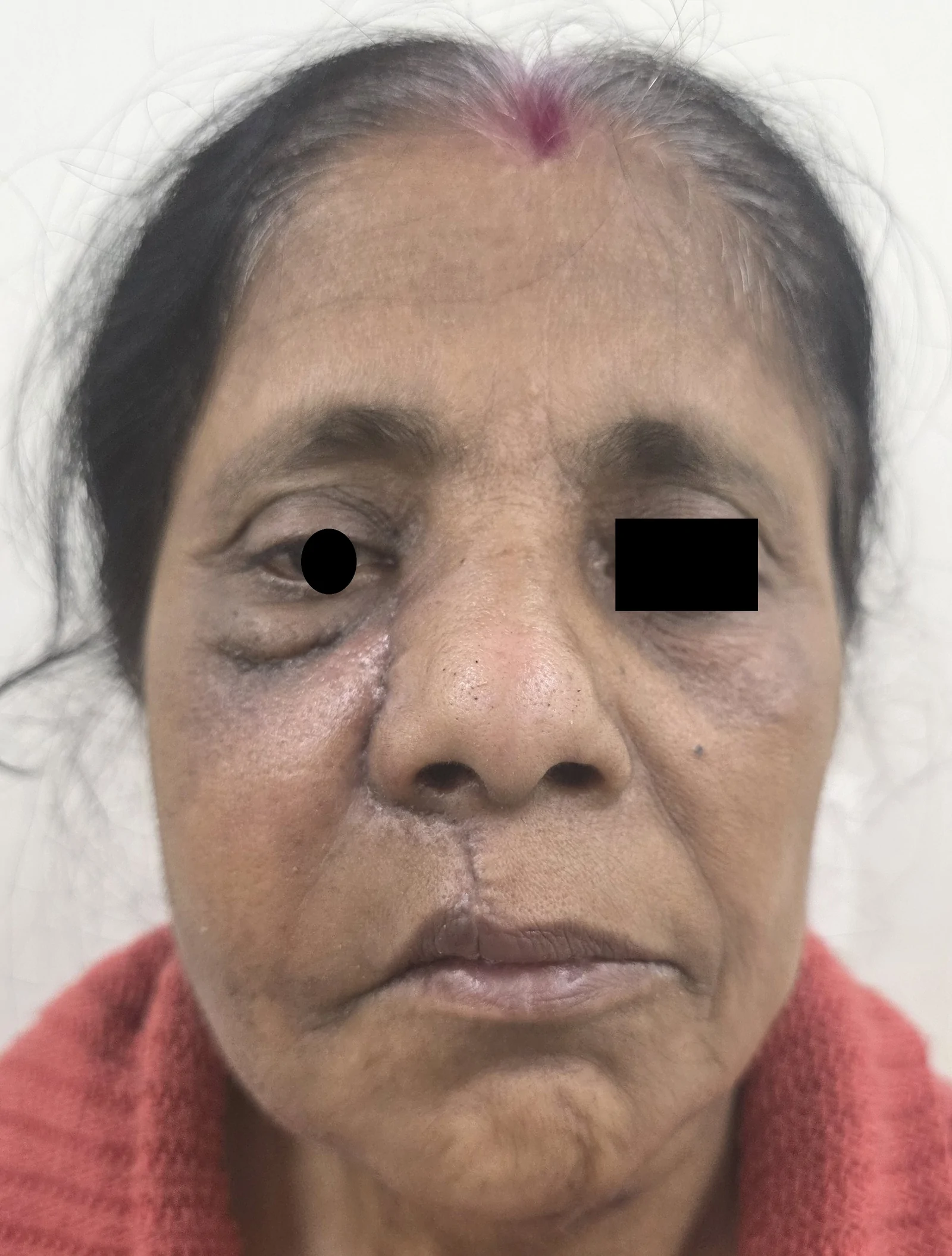
Post-operative result.

## Discussion

Several authors have created systematic classification schemes for midface defects. In our opinion, the most pertinent and descriptive classification for orbitomaxillary defects remains that put forth by Brown and Shaw (6). In their original description, maxillectomy defects were classified into four vertical classes (I to IV) and three horizontal classes (a to c) (7). To include orbitomaxillary and nasomaxillary defects, Brown and Shaw modified the classification system to its current version which included six vertical classes (I to VI) and four horizontal classes (a to d) (6). In their classification, class 3 defects are total maxillectomy with globe preservation, along with horizontal defects ranging from a to d. Class 4 defects include total maxillectomy with orbital exenteration, class 5 defects are orbitomaxillary defects, and class 6 nasomaxillary defects.

In our study, we have included type 3–6 maxillectomy defects, which require primary reconstruction by free tissue transfer. Obturators, though a modality of reconstruction, are suited to patients with smaller defects that do not involve the orbit or facial skin, who have good dexterity, without visual impairment, without trismus, and who do not require postoperative radiotherapy (8).

There are two main types of chimeric flaps: intrinsic and fabricated chimeric flaps. Intrinsic chimeric flaps make use of the natural inherent vascular anatomy, and all flap components are dissected on their individual vascular leash, which converge to a single pedicle. Fabricated chimeric flaps involve selecting ideal components and performing intraflap microvascular anastomosis to create an ideal single chimeric flap (9). In our series, all 11 flaps were chimeric flaps. A total of 10 (91%) were intrinsic chimeric flaps, and one was a fabricated chimeric flap, which was based on the scapular system.

A chimeric flap has the advantage of providing multiple well-vascularized components with an independent degree of movement relative to each other, leading to ease of inset, lesser stretch on the paddle, and better approximation of the edges to provide a watertight seal. This facilitates better healing and less morbidity (10). For maxilloalveolar defects, the use of chimeric flaps, compared with conventional flaps, results in fewer complications, including oronasal fistula and wound breakdown, and a shorter hospital stay (10).

The indications for exenteration in sinonasal malignancy include involvement of the orbital apex, full-thickness invasion through the periorbita into retrobulbar fat, extension into the extraocular eye muscles, and invasion of the bulbar conjunctiva or sclera (11). Reconstruction of an orbital exenteration defect associated with maxillectomy, with or without skull base resection, is a challenge and is conventionally performed using a soft tissue flap. However, such complex orbitomaxillary defects are multicomponent and include a palatal lining defect for oronasal separation, an external skin defect for advanced tumors, loss of orbital walls with exenteration, which results in a high-volume defect, and a skull base defect when combined with craniofacial resection (12).

The reconstructive goals in such patients are to achieve adequate cranio-nasal separation, oro-nasal separation, facial contour, skin cover, and orbital and dental rehabilitation. Ocular rehabilitation of such patients is a challenge because of the loss of the eyelids and orbital floor and the absence of an orbital cavity lined by epithelium and an osteointegrated implant to stabilize the prosthesis. This precludes the use of orbital or ocular prosthesis. Most of these patients opt for spectacle-mounted prosthesis (12).

The endpoint of orbital exenteration reconstruction is placement of epithesis within a thin free flap adherent to the bony orbital margin. This necessitates collaboration with the prosthetist from the time of primary surgery to plan the optimal socket shape for further prosthetic rehabilitation (13). Exenterated patients need a concave, neo-orbital socket contour, with early planning for anaplastology. In extensive orbital exenterations, where immediate flap shaping into a concave form is not feasible, flap debulking should be planned after completion of treatment, along with placement of osteointegrated implants for prosthetic rehabilitation (13).

In our series, six patients had exenteration. Five out of the six patients had a type 5 maxillectomy defect, and two patients had a simultaneous craniofacial resection. All of the patients were reconstructed with chimeric soft tissue flaps. In five patients, anterolateral thigh flap was used, and in one patient chimeric LD flap was used. The flaps were designed according to the defect, and there was no vascular compromise and they healed well.

In patients with type 3 total maxillectomy, the inferior orbital rim and orbital floor are resected. They warrant accurate reconstruction to maintain orbital volume and upper facial projection. Maintaining accurate orbital volume prevents proptosis, enophthalmos, and inferior migration of the globe (14).

Fibula remains the gold standard for reconstruction of total maxillectomy defects (15). The advantage is long pedicle length with large diameter vessels and a triangular bone which can be osteotomized into multiple components and can be used in different configurations to reconstruct the alveolar and the infraorbital buttress, and the mesh can be used to reconstruct the orbital floor and fixed over the fibula construct. However, it has its disadvantages of donor site morbidity, limited soft tissue for volume correction, and the chance of torsion of the skin perforator when used for a total maxillectomy defect with multiple bone struts. As the fibula bone lacks height, different bone struts are required for the alveolar part and the infraorbital rim part, and due to the reduced width, a mesh is required to reconstruct the orbital floor. Titanium mesh is one of the commonly used implants to repair orbital wall defects because it is highly biocompatible and readily shaped into the defects (15).

The subscapular free flaps system has been well established to provide the most generous source of soft tissue along with bone components. Angular branch-based chimeric scapular osteomuscular flaps were a major evolution of the classic circumflex scapular artery-based flaps, with the added advantage of a long pedicle (16). The advantage of the scapula flap is the large amount of soft tissue component that can be harvested, with the donor area being closed primarily. There is minimal shoulder dysfunction, with the flap being an ideal choice for elderly patients with documented or impending peripheral vascular disease when compared to a fibula flap. The bone itself does not need multiple osteotomies to contour according to the defect, and it has the propensity to be designed as a chimeric flap with multiple components as per the defect requirement. However, it has its disadvantages, the most significant being change in position while harvesting, though the angular branch-based flap can be harvested in the supine position (16, 17).

In our series, four (36%) patients had total maxillectomy with globe preservation. All of them were reconstructed with chimeric bone flaps. Three underwent a chimeric fibula flap, and one underwent chimeric scapula flap. The bone segments, as well as the contouring of the titanium mesh for the orbital floor, were designed based on the 3D model. Titanium mesh was used on all three patients requiring a fibula flap. For the patient with a chimeric scapula flap, the angular bone was contoured to the floor defect, and it did not require a mesh. There were no vascular complications. At 3 months post-treatment, no patient had enophthalmos or diplopia. There were no implant-related complications.

Over the past decade, significant advances have been made in preoperative planning and, specifically, the use of 3D modelling. For complex maxillary reconstructions, preoperative 3D planning can allow for more precise intraoperative bone cuts and improved orientation of the pedicle. These can together improve overall intraoperative efficiency, shorten operative times, and potentially further minimize risks to patients (18). However, these benefits should be weighed against the potential for increased costs and a delay to surgery to allow for prefabricated models to be created. Computer-aided techniques such as virtual planning, 3D printed model, and pre-bending guide template can be used for the harvesting and positioning of a free fibula flap, the forming of personalized titanium mesh, and ultimately the improvement of clinical efficacy of maxillary and orbital floor reconstruction. In their study, it was used in six patients with total maxillectomy defect (18).

In all of our patients with type 3 total maxillectomy, a 3D printed model was created, and the bone struts as well as the contour of the titanium mesh were done based on the model.

Fu et al. had used a 3D resin model for pre-bent templates to guide the harvesting and positioning of the fibular flap during surgery. Based on the model, the titanium mesh was contoured and used to reconstruct the orbital floor defect (15). Avraham et al. found that patients receiving CAD-CAM-based reconstructions had increased rates of dental rehabilitation and experienced minimized operative time (19).

For orbital reconstruction, a revision procedure was needed for necrosis and/or infection of the costal cartilage graft in eight (17%) cases (17). For 47 (55.9%) patients, the orbital floor was reconstructed using a generally costal cartilage graft (17). However, they had used non-vascularized rib for orbital floor reconstruction.

Free flap loss is uncommon and estimated to be around 2%–5%; there is also an estimated 6% rate of partial flap loss (20). In our study, there were no flap re-explorations and no partial or complete flap loss. A meta-analysis including 805 flaps was conducted by F Maglitto et al. to estimate the overall incidence of early complications. The pooled infection rate was 7%, the fistula rate was 12%, and the wound dehiscence rate was 16% (21). They concluded that these adverse events occurred several times more often than total or partial flap loss and accounted for the majority of reported complications (21).

In their study of fibula flap for maxillary reconstruction, Cho et al. reported an operative take-back rate of 19.2%. Total flap loss was 2.7%. Wound dehiscence was 13.5%. The plate exposure rate was 11%, and the rate of palatal fistula was 5.5%. No patient, however, had diplopia or enophthalmos. They had used CAD-CAM (22).

One of the three patients who underwent craniofacial resection had a CSF leak which settled conservatively. One patient had marginal skin necrosis of the skin island, which healed with regular dressings. No patient had a fistula.

Corederio et al. concluded that the outcomes after the reconstruction of the orbital floor were generally good; 24% of patients had either vertical or horizontal persistent diplopia after surgery, and nearly half of the patients had persistent ectropion, but no patient required further surgical interventions (23). In our study, in all patients planned for total maxillectomy, we used a 3D model to accurately design the bone strut and bending of the titanium mesh for the orbital floor. We did not see any diplopia nor enophthalmos, which was assessed at a minimum of 3 months after the completion of the entire treatment. All of our patients had unrestricted diet and near-normal speech.

However, this study has drawbacks. It is a retrospective study with a short follow-up after the completion of treatment. Long-term complications, namely, plate exposure, enophthalmos, and delayed volume loss of the flap post-radiotherapy, could not be assessed.

## Conclusion

Sinonasal malignancy may result in complex orbitomaxillary defects, which are three-dimensional. They require multiple component reconstruction, which are at different planes, and chimeric flaps with their multiple components suit well for such defects. For type 3 defects, osseous reconstruction is warranted and a chimeric osteo-cutaneous flap, with the aid of a 3D face model, helps in simplifying a complex reconstruction.

## Data Availability

The datasets presented in this article are not readily available because not used. Requests to access the datasets should be directed to KP dockarnav@gmail.com.
